# Dangerous Forms of Quackery

**Published:** 1863-10

**Authors:** 


DANGEROUS FORMS OF QUACKERY.—We have never known quackery to put on so bold a front as it does at this time. As an evidence of this, we have but to refer to the columns of our daily and weekly newspapers, and to the shelves of most of our drug stores. The former are burdened with advertisements of quack surgeons and quack doctors, and the latter are groaning with the weight of medicines of every description. Itinerant eye and ear surgeons (?) under assumed names, monopolize whole pages of our large city dailies with elaborate treatises on diseases of those tender organs, calculated to fix popular attention and entrap the unwary. The amount of pseudo-learning that they profess is only equaled by their brazen impudence, and their pretensions to skill by the irreparable damage they do to the sensitive organs of sight and hearing of their dupes.

But extensive as are the evils connected with the operations of these traveling oculists and aurists with the hard names, all of them professing to be students of European surgeons of eminence, who in all probability never saw or heard of them, they may merely involve the loss of the sense of sight or of hearing. Bad as that is, there is a worse form of quackery prevalent now; one which, if we may judge by the various forms it takes, and the extent to which the advertising is carried on in the newspapers, must receive a very heavy patronage at the hands of the public. We refer to the various forms of "Bitters" which are so extensively thrust down the throats of the community, and whose virtues are elaborately set forth in the daily and weekly newspapers, not excepting most of the *religious* papers of the day.

If these were the mere vile decoctions of bitter herbs that they profess to be, it would be a matter of small consequence. Little harm might be done. But in nine cases out of ten, these "Bitters" are nothing more or less than alcoholic liquors in some form, principally gin and whisky. The immense consumption of these alcoholic "bitters" by the community is perfectly astounding, and the amount of evil that their use is laying up in store for the rising generation, in begetting a taste for the inebriating cup, is beyond computation. In the sale of these vile alcoholic compounds, almost every drug and grocery store in the land is in reality a dram shop, without the formality of taking out a license and paying a tax to ease their consciences!

This evil has grown to such a magnitude that it is high time that legal restraints were thrown around the sale of these vile "bitters." These nostrum venders are putting a poisoned chalice to the lips of our sons and our daughters, and the crop that will be reaped by the community will be a harvest of drunkards and vagabonds to its utter confusion and disgrace, and the burdening of our almshouses and prisons, and the consequent increase of our taxes for their support. If our druggists and grocers are going to be allowed to sell gin and whisky by the quart, under the name of "Bitters," let us have the poor consolation of knowing that they pay an extra tax for the privilege, as tavern keepers do.

Let our profession lose no opportunity of warning the community against these villainous compounds particularly, as well as against all forms of quackery.—*Med. & Surg. Reporter.*

Singular effect of Fright.—A very interesting fact in a physiological point of view, has just taken place at Kenezew, in the Palatinate of Plock. A detachment of one hundred Cossacks had just invaded the village, and were preparing to pillage the chateau. At the same moment M'me. Wiwerska, wife of an ex-colonel of the Polish army, and mother-in-law of the proprietor, M. Wosinoki, was apparently to breathe her last, and the family, with the death wax-light in their hands, surrounded her bed. At the cry of the Cossacks, who had come to lay hold of her son-in-law, the dying woman sat up, then got out of bed, and, with the most perfect presence of mind, gave such orders as were necessary under the circumstances. The danger to which her children were exposed re-animated her departing spirit. She is still alive, but has frequent hysterical attacks.—*Med. & Surg. Reporter.*

